# The Expression and Clinical Significance of *C1orf106* in Low‐Grade Serous Ovarian Cancer

**DOI:** 10.1111/jog.70118

**Published:** 2025-10-23

**Authors:** Feifei Song, Xiaojing Chen, Tao Zhang, Xuedong Tang, Xiaodong Cheng

**Affiliations:** ^1^ Department of Gynecologic Oncology Women's Hospital, School of Medicine, Zhejiang University Hangzhou Zhejiang China; ^2^ Zhejiang Provincial Key Laboratory of Precision Diagnosis and Therapy for Major Gynecological Diseases Women's Hospital, School of Medicine, Zhejiang University Hangzhou Zhejiang China; ^3^ Department of Obstetrics and Gynecology Jiaxing University Affiliated Women and Children Hospital Jiaxing Zhejiang China

**Keywords:** bioinformatics, *C1orf106*, INAVA, low‐grade serous ovarian cancer, survival analysis

## Abstract

**Aim:**

Low‐grade serous ovarian cancer (LGSOC) is a rare subtype of ovarian cancer with distinct biological behavior. This study aimed to identify new biomarkers with potential diagnostic and prognostic value for LGSOC.

**Methods:**

Gene‐expression data were downloaded from the Gene Expression Omnibus (GEO). Differentially expressed genes (DEGs) were identified using R. Functional enrichment analyses were conducted to determine the biological functions and signaling pathways associated with DEGs. The mitogen‐activated protein kinase (MAPK) pathway‐related gene, chromosome 1 open reading frame 106 (*C1orf106*), was selected as the target gene. Immunohistochemistry and quantitative real‐time polymerase chain reaction (qRT‐PCR) were performed to verify its expression. Associations between *C1orf106* expression and the clinical features of patients were analyzed using the chi‐square (*χ* ^2^) test. Prognostic significance was evaluated with survival analyses.

**Results:**

A total of 3099 upregulated and 4968 downregulated genes were identified in LGSOC. Gene set enrichment analysis (GSEA) demonstrated significant alterations in KRAS signaling and metabolic pathways between LGSOC and healthy controls. Kyoto Encyclopedia of Genes and Genomes (KEGG) and Gene Ontology (GO) analyses revealed enrichment in immune response and MAPK pathway alterations. Immunohistochemistry and qRT‐PCR confirmed that *C1orf106* expression in LGSOC tissues was significantly higher than in normal ovarian tissues. Clinically, high *C1orf106* expression was associated with lower BMI (< 25 kg/m^2^), the absence of visible residual disease, and improved progression‐free survival (PFS) and overall survival (OS) in univariate Cox and Kaplan–Meier analyses.

**Conclusions:**

*C1orf106* may serve as a promising marker for the diagnosis and prognosis of LGSOC.

## 1 Introduction

Ovarian cancer is the most lethal gynecologic malignancy. The recurrence or metastasis rate in patients with ovarian cancer (OC) is approximately 70% within 3 years, and the five‐year survival rate for advanced OC is only about 30% [1]. Low‐grade serous ovarian cancer (LGSOC) is a relatively rare subtype, accounting for approximately 2% of all epithelial ovarian cancers [2]. Compared with patients who have high‐grade serous ovarian cancer (HGSOC), those with LGSOC are typically about 10 years younger [3, 4]. In addition, patients with LGSOC generally present with lower volumes of ascites and lower pretreatment cancer antigen 125 (CA‐125) levels than patients with HGSOC [5]. Estrogen receptor (ER) expression is detected in the majority of LGSOC cases (50%–90%), whereas progesterone receptor (PR) expression is observed in 40%–50%. These findings provide the rationale for hormone therapy in LGSOC [6]. Although the prognosis of LGSOC is generally better than that of HGSOC [7], its inherent resistance to chemotherapy [8, 9] still poses major treatment challenges.

The mechanisms underlying LGSOC development remain unclear. The prevailing hypothesis suggests a stepwise progression from benign serous cystadenoma to serous borderline tumor (SBT), micropapillary SBT, and ultimately to invasive LGSOC [2]. At the molecular level, frequent activation of the mitogen‐activated protein kinase (MAPK) pathway has been reported in LGSOC [10, 11]. For example, BRAF mutations are observed more often in LGSOC (2%–33%) than in HGSOC (approximately 9%) [12]. Interestingly, BRAF alterations usually indicate a more favorable prognosis in LGSOC [13], providing a rationale for the use of BRAF and MEK inhibitors. Currently, the standard treatment for LGSOC mirrors that of HGSOC and includes surgical debulking followed by platinum/taxane‐based chemotherapy [13]. However, because LGSOC is frequently diagnosed at advanced stages and is relatively chemoresistant, the identification of novel biomarkers is crucial to improving diagnosis and prognosis.

In this study, we applied bioinformatics approaches to identify differentially expressed genes (DEGs) in LGSOC and normal ovarian tissues. Among these, chromosome 1 Open Reading Frame 106 (*C1orf106*), a MAPK‐related gene, was selected as the target gene for subsequent validation and analysis.

*C1orf106*, also known as innate immune activator (INAVA), is a protein‐coding gene located on chromosome 1 [14]. It participates in multiple biological processes, including the positive regulation of cytokines and intracellular signal transduction. *C1orf106* expression is typically downregulated in inflammatory bowel disease (IBD). Similarly, in dermatomyositis, where it promotes CD4+ T cell infiltration, *C1orf106* is expressed at low levels [15]. In contrast, *C1orf106* is highly expressed in several cancers. For instance, in lung adenocarcinoma across all stages, *C1orf106* is markedly upregulated and may contribute to tumor development [16]. It also enhances the aggressiveness of papillary thyroid cancer by upregulating *MMP9* expression [17]. In ovarian cancer, *C1orf106* has been reported to promote cell migration [18]; however, its role in LGSOC remains undefined.

Based on previous studies, we evaluated *C1orf106* expression using immunohistochemistry (IHC) and quantitative real‐time polymerase chain reaction (qRT‐PCR). We further analyzed the association between *C1orf106* expression and clinicopathological features in patients with LGSOC and assessed its prognostic significance. In summary, this study provides new evidence on the pathogenesis and progression of LGSOC.

## 2 Materials and Methods

### 2.1 Database and Bioinformatics Analysis

#### 2.1.1 Differentially Expressed Genes (DEGs) Analysis

The GSE14001 dataset, containing messenger RNA (mRNA) expression data from LGSOC samples (*n* = 10) and normal human ovarian surface epithelia (*n* = 3), was downloaded from the GEO database. DEGs between LGSOC and control groups were identified using the “limma” package in R. Genes with |log_2_ fold change (FC)| > 1 and adjusted *p* < 0.05 were considered significant. Volcano plots were generated with the gplots package, and heatmaps were created using OmicStudio (https://www.omicstudio.cn).

#### 2.1.2 Gene Enrichment Analysis

Hallmark gene set enrichment analysis (GSEA) was performed using OmicStudio tools to evaluate differentially enriched pathways in LGSOC versus normal controls. Significant enrichment was defined as nominal *p* < 0.05 and false discovery rate (FDR) < 0.05. DEGs were uploaded to the DAVID platform (https://david.ncifcrf.gov/) for Gene Ontology (GO) and Kyoto Encyclopedia of Genes and Genomes (KEGG) analyses, with *p* < 0.05 and FDR < 0.05 as thresholds. Histograms were generated using OmicStudio, and bubble plots were created using GraphBio (http://www.graphbio1.com/).

### 2.2 Protein–Protein Interaction (PPI) Networks

PPIs were predicted using the STRING database (https://string‐db.org/) to assess potential functional relationships. Proteins encoded by the top 200 DEGs were uploaded, with the confidence‐score threshold set at > 0.4. PPI networks were constructed in Cytoscape with the following parameters: degree cutoff = 2, node score cutoff = 0.2, k‐score = 2, and maximum depth = 100.

### 2.3 Selection of the Target Gene

Because MAPK pathway mutations frequently occur in LGSOC [19], we focused on MAPK‐related pathways in the GO analysis. The intersection between the top 30 DEGs and MAPK pathway–associated genes was examined to select the most significant MAPK‐related DEG in LGSOC for further analysis.

### 2.4 Clinical Data Collection and Analysis

#### 2.4.1 Patients

Paraffin‐embedded tissue sections and clinical data of patients with LGSOC were collected from the Women's Hospital, School of Medicine, Zhejiang University. The inclusion criteria were: (I) Age: 18–80 years; (II) Diagnosis between January 1, 2010, and December 23, 2021; (III) Postoperative pathological confirmation of LGSOC; and (IV) Availability of complete clinical data.

The exclusion criteria were: (I) Unclear pathological diagnosis; (II) History of other malignant diseases; and (III) Incomplete clinical information. A total of 52 eligible LGSOC tissues were included. Additionally, 50 normal ovarian tissues were obtained for RNA extraction and immunohistochemistry. This study was approved by the Ethics Committee of the Women's Hospital, School of Medicine, Zhejiang University (Approval No. IRB‐20220029‐R).

#### 2.4.2 Clinicopathological Data and Follow‐Up

Clinicopathological variables potentially affecting prognosis were collected, including age [20], body mass index (BMI) [21], maximum tumor diameter [22], tumor laterality (unilateral or bilateral) [22], preoperative serum CA‐125 level [23], ascitic fluid volume [24, 25], cytology of ascitic fluid [26], International Federation of Gynecology and Obstetrics (FIGO) stage [27], ER expression [6], PR expression [6], residual disease status^27^, and chemotherapy response [28, 29]. Follow‐up data were obtained through the institutional database. Endpoints included progression‐free survival (PFS), overall survival (OS), and survival status. The follow‐up period extended to March 2022, with a median follow‐up of 62 months (range: 4–128 months). At the last follow‐up, 32 patients had no recurrence, 20 experienced recurrence, and 13 had died of disease. PFS was defined as the interval between the date of surgery and recurrence. OS was defined as the interval between the date of surgery and death or last contact.

### 2.5 Immunohistochemistry

Paraffin‐embedded tissue sections were deparaffinized, and antigen retrieval was performed using a citric acid buffer. Sections were blocked with 3% bovine serum albumin (BSA) at room temperature, followed by incubation with primary antibodies against *C1orf106* (21506‐1‐AP, Proteintech, 1:800 dilution) overnight at 4°C. After washing, the slides were incubated with secondary antibodies for 30 min. Diaminobenzidine (DAB) solution was used for color development, which was monitored microscopically. When the reaction produced a brownish‐yellow color, development was terminated by rinsing with water, and the sections were sealed with neutral resin. Slides were examined and photographed under a microscope, and staining intensity was evaluated semi‐quantitatively.

Staining intensity was scored as: 0 = negative, 1 = weak, 2 = moderate, and 3 = strong. The percentage of positive cells was scored as follows: 0 = no positive cells; 1 = ≤ 10% positive cells; 2 = 11%–50% positive cells; 3 = 51%–80% positive cells; 4 = 81%–100% positive cells. *C1orf106* expression was assessed using an immunoreactive score (IRS), calculated by multiplying the intensity score by the percentage score. A total score of < 3 was defined as low expression, while a score ≥ 3 was defined as high expression [30].

### 2.6 qRT‐PCR

Ten LGSOC tissues and ten normal ovarian tissues (collected between January 1, 2019, and December 23, 2021) were used for RNA extraction. RNA was isolated using the RNAPREP PURE Total RNA Extraction Kit for Paraffin‐embedded Tissue Sections (Tiangen Biotech Co. Ltd., Beijing, China). Complementary DNA (cDNA) was synthesized from 1 μg of total RNA using the HiScript III RT SuperMix for qPCR (with genomic DNA [gDNA] wiper) (Vazyme Co. Ltd., Nanjing, China). qRT‐PCR was performed with ChamQ SYBR qPCR Master Mix (Without ROX) (Vazyme Co. Ltd., Nanjing, China).

Relative mRNA expression levels were calculated using the 2^−ΔΔCt^ method. The primers used were: *C1orf106*: forward, GAGGAATCCCAAGTGCCAAAA and reverse, GGGCTTCTCATAGGGGTGGT; GAPDH forward, ACAACTTTGGTATCGTGGAAGG and reverse, GCCATCACGCCACAGTTTC.

### 2.7 Statistical Analysis

Statistical analyses were performed using SPSS, version 31.0. Associations between *C1orf106* expression and clinicopathological variables were examined using the *χ* ^2^ test. Prognostic factors identified in the univariate analysis were subsequently assessed by multivariate Cox regression analysis. Kaplan–Meier survival curves were generated with GraphPad Prism version 9.0 to evaluate the relationship between *C1orf106* expression and LGSOC prognosis. A *p*‐value < 0.05 was considered statistically significant.

## 3 Results

### 3.1 DEGs Between LGSOC and Normal Ovarian Tissues

**FIGURE 1:**
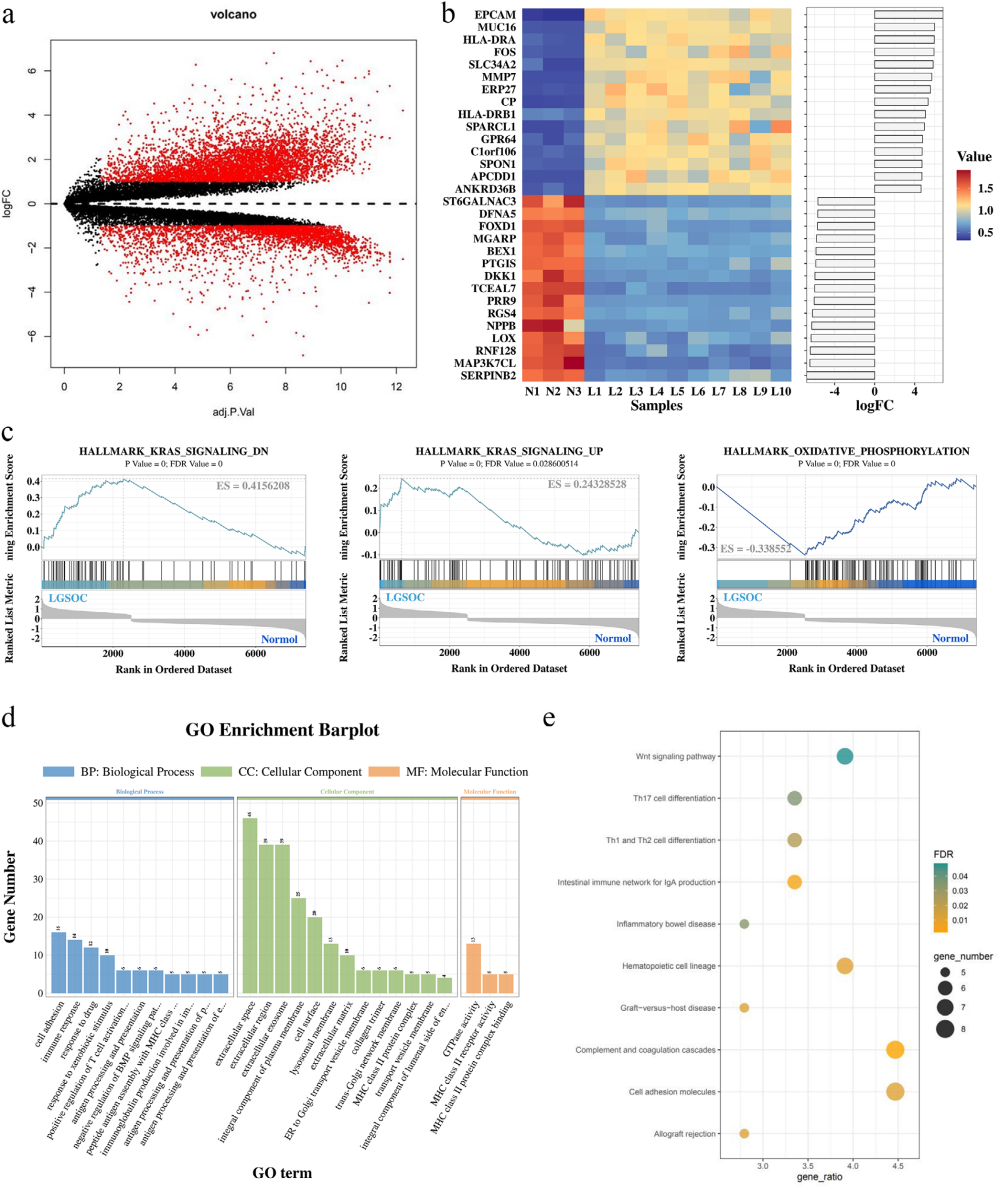
Differentially Expressed Genes (DEGs) and Functional Enrichment Analysis in Low‐Grade Serous Ovarian Cancer (LGSOC). (a) Volcano plot of DEGs between LGSOC (*n* = 10) and normal human ovarian surface epithelia (NOSE; *n* = 3). (b) Heatmap of the top 30 DEGs (*N* = NOSE samples; L = LGSOC samples). (c) Gene set enrichment analysis (GSEA) demonstrated upregulation of KRAS‐associated hallmarks and downregulation of oxidative phosphorylation pathways. Normalized enrichment scores (NES), *p*‐values, and false discovery rates (FDR) are shown for each pathway. (d) Gene Ontology (GO) analysis revealed significant enrichment of immune‐related terms, including positive regulation of T cell activation, antigen processing and presentation, and major histocompatibility complex (MHC) class II receptor activity. (e) Kyoto Encyclopedia of Genes and Genomes (KEGG) pathway analysis identified enrichment of Wnt signaling, T helper 17 (Th17), T helper 1 (Th1), T helper 2 (Th2) cell differentiation, phagosome, cell adhesion molecules, allograft rejection and complement/coagulation cascades.

The GSE14001 dataset was obtained from the Gene Expression Omnibus (GEO), including samples from LGSOC (*n* = 10) and normal human ovarian surface epithelia (*n* = 3). Using the criteria of |log_2_FC| > 1 and adjusted *p* < 0.05, 3099 upregulated and 4968 downregulated genes were identified. A volcano plot of DEGs between LGSOC and normal ovarian tissues is shown in Figure 1a, and the expression levels of the top 30 DEGs are displayed in a heatmap (Figure 1b).

### 3.2 Functional Enrichment Analysis

Hallmark GSEA was performed to identify enriched pathways in patients with LGSOC samples compared with normal controls (Figure 1c). Sixteen pathways were significantly enriched (nominal *p* < 0.05, FDR < 0.05). Among these, HALLMARK_KRAS_SIGNALING_DN and HALLMARK_KRAS_SIGNALING_UP were upregulated in LGSOC, suggesting a possible role for KRAS mutations in LGSOC. Conversely, HALLMARK_OXIDATIVE_PHOSPHORYLATION was downregulated, indicating altered metabolic activity.

Subsequently, DEGs were submitted to the DAVID platform for GO and KEGG enrichment analyses. Significant GO terms (Figure 1d; *p* < 0.05, FDR < 0.05) were mainly enriched in pathways including positive regulation of T cell activation, MHC class II molecule–mediated immune response, antigen processing and presentation, drug response, cell adhesion, and negative regulation of BMP signaling. KEGG analysis identified 10 enriched signaling pathways (Figure 1e), such as complement and coagulation cascades, cell adhesion molecules, T cell differentiation, IgA production, and the Wnt signaling pathway. Collectively, these findings suggest that immune‐response activation and regulation play important roles in LGSOC.

### 3.3 PPI Network

**FIGURE 2:**
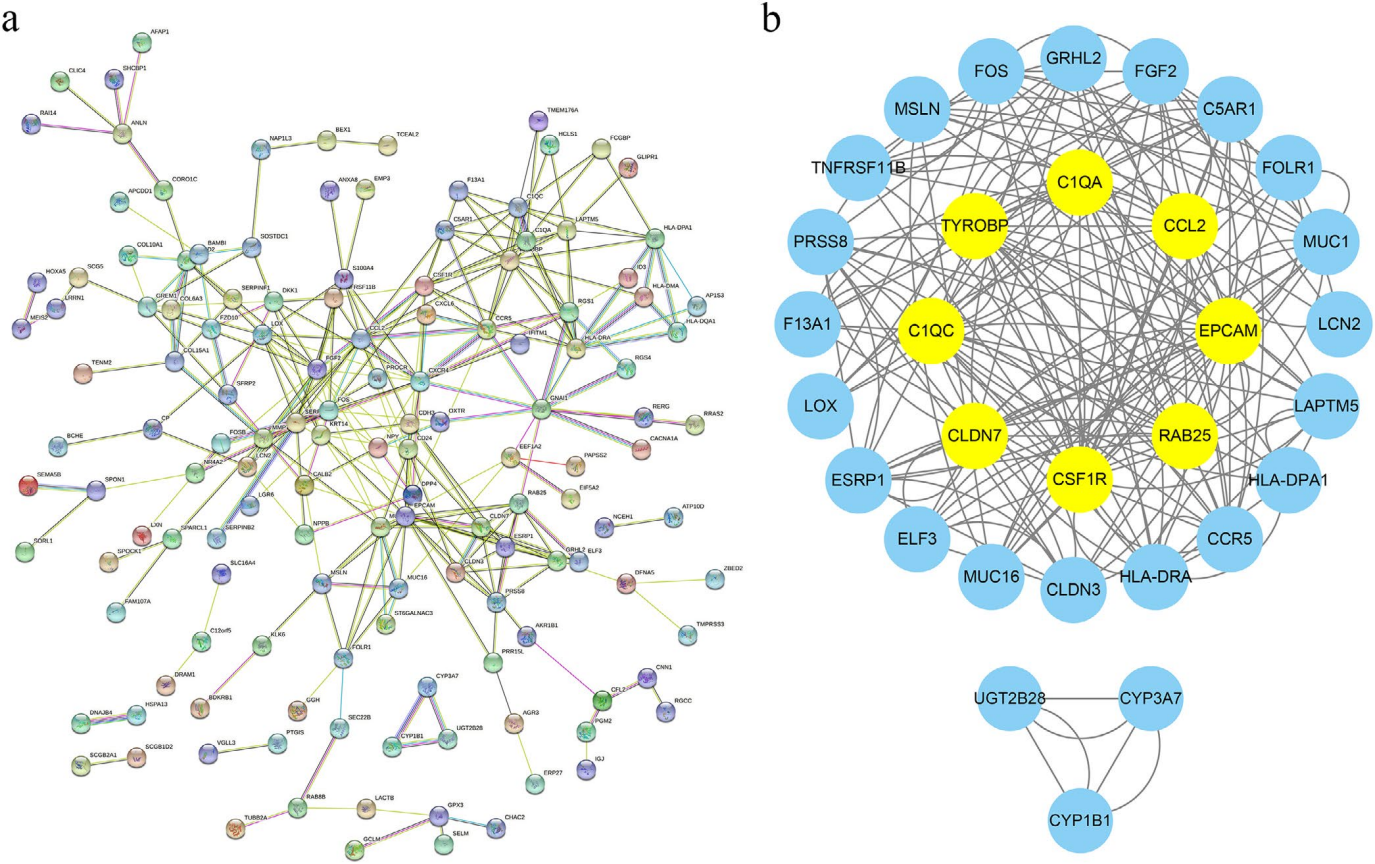
Protein–Protein Interaction (PPI) Network of Differentially Expressed Genes (DEGs). The PPI network was constructed to illustrate associations among DEGs between low‐grade serous ovarian cancer (LGSOC) and normal human ovarian surface epithelia (NOSE). Nodes represent proteins encoded by DEGs, and edges represent predicted protein–protein interactions.

The top 200 DEGs were analyzed using the STRING database to construct a PPI network. The resulting network contained 186 nodes and 290 edges, with an average node degree of 3.12 and a clustering coefficient of 0.443 (Figure 2a). The enrichment *p*‐value was < 1.0 × 10^−16^. Within this network, genes such as GRHL2, FGF2, C5AR1, FOLR1, MUC1, LCN2, LAPTM5, HLA‐DPA1, CCR5, HLA‐DRA, CLDN3, MUC16, ELF3, ESRP1, LOX, F13A1, PRSS8, TNFRSF11B, MSLN, FOS, C1QA, CCL2, EPCAM, RAB25, CSF1R, CLDN7, C1QC, and TYROBP exhibited significant mutual interactions (Figure 2b), suggesting central and cooperative roles in LGSOC development. In contrast, UGT2B28, CYP3A7, and CYP1B1 formed a smaller network, indicating possible independent or pathway‐specific functions.

### 3.4 Identification of *C1orf106* as the Target Gene

**FIGURE 3:**
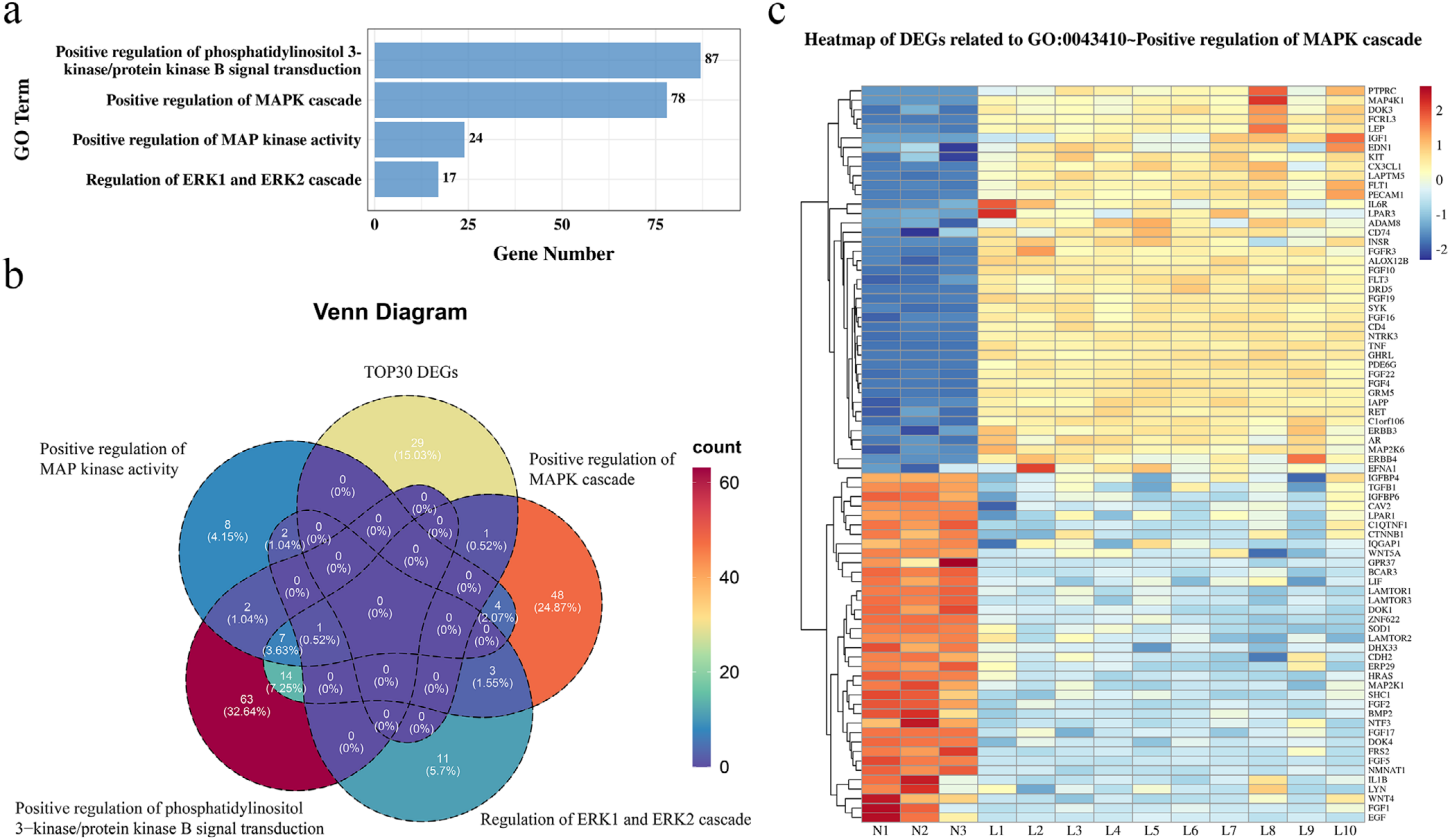
Identification of Chromosome 1 Open Reading Frame 106 (*C1orf106*) as a Candidate Target Gene. (a) Gene Ontology (GO) analysis revealed significant enrichment of mitogen‐activated protein kinase (MAPK) pathway–related terms. (b) Venn diagram showing the overlap between the top 30 differentially expressed genes (DEGs) and those enriched in MAPK‐related pathways. (c) Heatmap showing DEGs involved in GO:0043410 (positive regulation of the MAPK cascade).

Because MAPK pathway mutations frequently occur in LGSOC [19, 31], we focused on MAPK‐related pathways in GO analysis. Four significant enriched pathways were identified (Figure 3a): GO:0043406 (positive regulation of MAP kinase activity), GO:0043410 (positive regulation of MAPK cascade), GO:0070372 (regulation of extracellular signal‐regulated kinase 1 [ERK1] and [ERK2] cascade), and GO:0051897 (positive regulation of phosphatidylinositol 3‐kinase/protein kinase B signaling). By intersecting the top 30 DEGs with genes from these pathways (Figure 3b), we identified a single overlapping gene, *C1orf106*, within GO:0043410 (positive regulation of MAPK cascade). Heatmap visualization confirmed its differential expression (Figure 3c).

Functional annotation of *C1orf106* (https://www.ncbi.nlm.nih.gov/gene/55765) showed that, in addition to its association with MAPK signaling, it was linked to immune processes, including immune system regulation, cytokine production, and positive regulation of interleukin‐1β production. These results are consistent with our enrichment analysis, which indicated that DEGs in LGSOC were predominantly enriched in immune‐related pathways.

Therefore, *C1orf106* was selected as the target gene for further investigation.

### 3.5 mRNA and Protein Expression of *C1orf106* in LGSOC and Normal Ovarian Tissues

**FIGURE 4:**
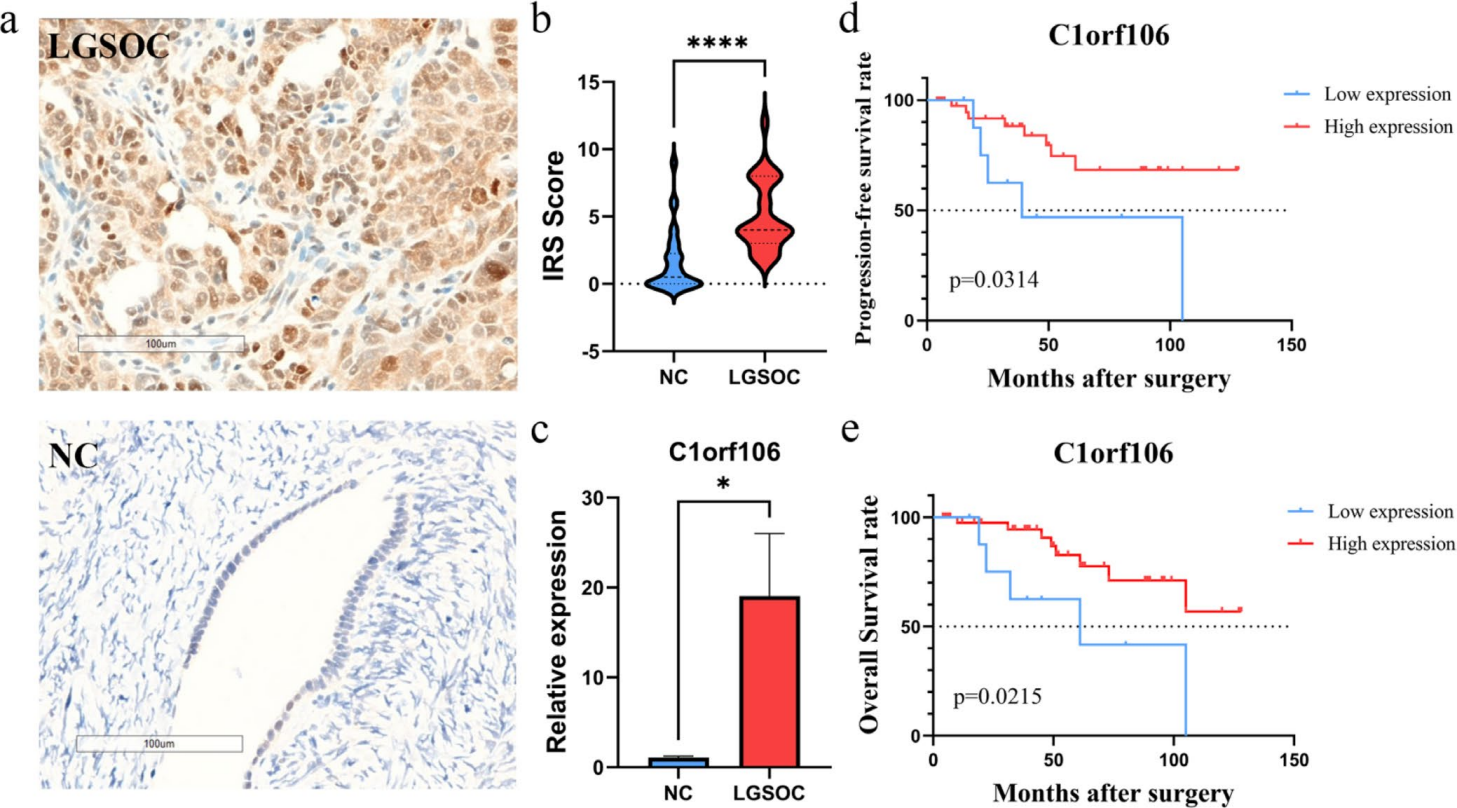
Clinical Significance of Chromosome 1 Open Reading Frame 106 (*C1orf106*) Expression in Low‐Grade Serous Ovarian Cancer (LGSOC). (a) Representative immunohistochemical (IHC) staining (×400 magnification) and quantitative reverse transcription polymerase chain reaction (qRT‐PCR) results comparing LGSOC and normal ovarian tissues. (b) Violin plots showing immunoreactive scores (IRS) of LGSOC (*n* = 52) and normal ovarian tissues (*n* = 50). (c) Relative *C1orf106* messenger RNA (mRNA) expression levels in LGSOC versus normal tissues, as determined by qRT‐PCR (*p* < 0.05). (d) Kaplan–Meier survival curves for progression‐free survival (PFS) stratified by *C1orf106* expression. (e) Kaplan–Meier survival curves for overall survival (OS) stratified by *C1orf106* expression.

Representative images of *C1orf106* expression in LGSOC tissues (*n* = 52) and normal ovarian tissues (*n* = 50) are shown in Figure 4a. Protein expression levels of *C1orf106* in LGSOC were significantly higher than those in normal ovarian tissues (*p* < 0.001) (Figure 4b). Consistent with these findings, qRT‐PCR analysis revealed that mRNA levels of *C1orf106* were significantly elevated in LGSOC compared with normal tissues (Figure 4c).

### 3.6 Associations Between *C1orf106* Protein Expression and Clinicopathological Features

Fifty‐two patients with LGSOC were included in the clinicopathological analysis. Their baseline characteristics are summarized in Table 1. Based on immunohistochemistry (IHC) scores, patients were classified into low‐expression (9/52, 17.31%) and high‐expression groups (43/52, 82.69%).

**TABLE 1:** Associations between C1orf106 protein expression and clinicopathological features.

Characteristics	*n* (*n* = 52)	C1orf106		χ^2^‐value	*p*
		Low (*n* = 9)	High (*n* = 43)		
Age at surgery, years					
< 45	25	5 (20.00%)	20 (80.00%)	0.016	0.899
≥ 45	27	4 (14.81%)	23 (85.19%)		
Body mass index, kg/m^2^					
< 25	32	2 (6.25%)	30 (93.75%)	6.250	*0.012*
≥ 25	18	7 (38.89%)	11 (61.11%)		
Unknown	2	0 (0.00%)	2 (100.00%)		
Maximum tumor diameter, cm					
< 10	23	2 (8.70%)	21 (91.30%)	1.324	0.250
≥ 10	28	7 (25.00%)	21 (75.00%)		
Unknown	1	0 (0.00%)	1 (100.00%)		
Tumor laterality					
Unilateral	16	3 (18.75%)	13 (81.25%)	0.000	1.000
Bilateral	36	6 (16.67%)	30 (83.33%)		
Preoperative serum CA125 level, U/mL					
< 35	14	2 (14.29%)	12 (85.71%)	0.000	0.987
≥ 35	36	7 (19.44%)	29 (80.56%)		
Unknown	2	0 (0.00%)	2 (100.00%)		
Ascitic fluid volume, mL					
≤ 100	36	5 (13.89%)	31 (86.11%)	0.646	0.422
> 100	14	4 (28.57%)	10 (71.43%)		
Unknown	2	0 (0.00%)	2 (100.00%)		
Cytology of ascitic fluid					
Negative	26	4 (15.38%)	22 (84.62%)	0.127	0.721
Positive	21	5 (23.81%)	16 (76.19%)		
Unknown	5	0 (0.00%)	5 (100.00%)		
FIGO stage					
I + II	24	2 (8.33%)	22 (91.67%)	1.479	0.224
III + IV	28	7 (25.00%)	21 (75.00%)		
PR expression					
≤ (++)	41	8 (19.51%)	33 (80.49%)	0.013	0.980
> (++)	9	1 (11.11%)	8 (88.89%)		
Unknown	2	0 (0.00%)	2 (100.00%)		
ER expression					
≤ (++)	26	5 (19.23%)	21 (80.77%)	0.000	1.000
> (++)	24	4 (16.67%)	20 (83.33%)		
Unknown	2	0 (0.00%)	2 (100.00%)		
Residual disease status					
Absence of residual disease	41	4 (9.76%)	37 (90.24%)	6.556	*0.010*
Visible residual disease	7	4 (57.14%)	3 (42.86%)		
Unknown	4	1 (25.00%)	3 (75.00%)		

Abbreviations: CA125:cancerCA125, cancer antigen 125; ER expression, estrogen receptor expression; FIGO stage:Internationalstage, International Federation of Gynecology and Obstetrics stage; PR expression, progesterone receptor expression; *p*‐value, Statistical significance, with values < 0.05 considered significant.

High *C1orf106* expression was significantly associated with lower BMI < 25 kg/m^2^ and absence of visible residual disease (*p* < 0.05). No significant associations were observed with age, tumor size, laterality (unilateral vs. bilateral), preoperative CA‐125 levels, ascitic fluid volume, cytologic examination of ascites, or ER/PR expression (*p* > 0.05).

### 3.7 Prognostic Value of *C1orf106* Expression in LGSOC

#### 3.7.1 The Relationship Between Clinicopathological Characteristics and PFS

Univariate analysis demonstrated that large ascitic fluid volume (> 100 mL), positive ascitic cytology, advanced FIGO stage (III–IV), visible residual disease, and low *C1orf106* expression were associated with shorter PFS (*p* < 0.05). Multivariate Cox regression analysis identified advanced FIGO stage (III–IV) as the only independent prognostic factor for PFS (Table 2).

**TABLE 2:** Univariate and multivariate analysis of progression‐free survival in patients with LGSOC.

Characteristics	Univariate analysis			Multivariate analysis		
	HR	95% CI	*p*	HR	95% CI	*p*
Age at surgery, years	0.944	0.363–2.455	0.907			
Body mass index, kg/m^2^	2.237	0.856–5.848	0.100			
Maximum tumor diameter, cm	1.985	0.644–6.120	0.233			
Tumor laterality	0.330	0.075–1.455	0.143			
Preoperative CA125, U/mL	4.990	0.659–37.803	0.120			
Ascitic fluid volume, mL	8.341	2.873–24.215	*< 0.001*	/	/	0.230
Cytology of ascitic fluid	4.994	1.742–14.318	*0.003*	/	/	0.341
FIGO stage	29.128	3.765–225.366	*0.001*	29.128	3.765–225.366	*0.001*
PR expression	1.013	0.330–3.112	0.982			
ER expression	0.515	0.146–1.815	0.320			
Residual disease status	2.934	1.067–8.068	*0.037*	/	/	0.630
C1orf106 expression	0.310	0.113–0.847	*0.022*	/	/	0.522

Abbreviations: CA125:cancerCA125, cancer antigen 125; ER expression, estrogen receptor expression; FIGO stage:Internationalstage, International Federation of Gynecology and Obstetrics stage; PR expression, progesterone receptor expression; *p*‐value, statistical significance, with values < 0.05 considered significant.

Kaplan–Meier survival analysis showed that patients with low *C1orf106* expression had a shorter median PFS (39 months) than those with high expression (*p* = 0.0314) (Figure 4d).

#### 3.7.2 The Relationship Between Clinicopathological Characteristics and OS

Univariate Cox regression demonstrated that advanced FIGO stage (III–IV), large ascitic fluid volume (> 100 mL), positive ascitic cytology, and low *C1orf106* expression were adverse prognostic factors for OS (*p* < 0.05). Multivariate Cox regression confirmed that advanced FIGO stage (III–IV) was the only independent prognostic factor (Table 3).

**TABLE 3:** Univariate and multivariate analysis of ovarall survival in patients with LGSOC.

Characteristics	Univariate analysis			Multivariate analysis		
	HR	95% CI	*p*	HR	95% CI	*p*
Age at surgery, years	1.284	0.419–3.932	0.661			
Body mass index, kg/m^2^	1.289	0.421–3.948	0.656			
Maximum tumor diameter, cm	1.894	0.514–6.983	0.337			
Tumor laterality	0.446	0.098–2.023	0.295			
Preoperative CA125, U/mL	32.755	0.144–7466.463	0.208			
Ascitic fluid volume, mL	6.305	1.914–20.766	*0.002*	/	/	0.925
Cytology of ascitic fluid	6.213	1.701–22.697	*0.006*	/	/	0.735
FIGO stage	128.297	1.135–14500.147	*0.044*	128.297	1.135–14500.147	*0.044*
PR expression	1.195	0.327–4.365	0.787			
ER expression	0.534	0.115–2.478	0.423			
Residual disease status	2.614	0.848–8.062	0.094			
C1orf106 expression	0.293	0.095–0.898	*0.032*	/	/	0.597

Abbreviations: CA125:cancerCA125, cancer antigen 125; ER expression, estrogen receptor expression; FIGO stage:Internationalstage, International Federation of Gynecology and Obstetrics stage; PR expression, progesterone receptor expression; *p*‐value, Statistical significance, with values < 0.05 considered significant.

Kaplan–Meier analysis further demonstrated that patients with low *C1orf106* expression had significantly shorter OS compared with those with high expression (*p* = 0.0215) (Figure 4e).

## 4 Discussion

Ovarian cancer remains one of the most prevalent and lethal gynecologic malignancies. LGSOC is a rare histologic subtype with distinct molecular features and clinical behavior [9, 32]. Compared with HGSOC, LGSOC typically presents at a younger age, grows more slowly, and exhibits relative resistance to platinum‐based chemotherapy. Although the prognosis of LGSOC is generally better than that of HGSOC, the overall prognosis remains unsatisfactory, with 5‐ and 10‐year survival rates of approximately 69% and 56%, respectively [33]. To improve patient outcomes, it is critical to identify clinicopathological factors and molecular biomarkers that may aid prognostic evaluation and guide targeted therapies.

Poly (ADP‐ribose) polymerase inhibitors (PARPis) are widely used as maintenance therapy in ovarian cancer, particularly in patients with *BRCA1*, *BRCA2*, or homologous recombination deficiency (HRD) mutations [34]. Because MAPK pathway mutations are common in LGSOC, clinical research has explored targeted therapies involving BRAF and MEK inhibitors. For example, Farley et al. [35] conducted a phase II trial of selumetinib (a MEK1/2 inhibitor) in recurrent LGSOC and peritoneal carcinoma, reporting that 15% of patients achieved complete or partial responses and 65% experienced stable disease, suggesting selumetinib as a potential option for this patient population. Bedard et al. [36] later evaluated buparlisib (a pan‐PI3K inhibitor) in combination with trametinib (a MEK1/2 inhibitor) in *RAS‐* or *BRAF‐*mutant ovarian cancer, confirming notable antitumor activity. However, subsequent trials have shown that MEK inhibitors do not consistently benefit all patients with LGSOC [37, 38], underscoring the need for novel therapeutic targets.

This study analyzed mRNA expression data from the GEO database and identified 3099 upregulated and 4968 downregulated genes in LGSOC compared with normal ovarian tissues. GSEA demonstrated upregulation of KRAS signaling pathways, consistent with the known high frequency of *KRAS* mutations in LGSOC. Conversely, oxidative phosphorylation was downregulated, suggesting reduced aerobic respiration and increased reliance on anaerobic glycolysis. GO and KEGG enrichment analyses further indicated that the DEGs were mainly enriched in immune‐related pathways, such as T cell activation and MHC class II molecule–mediated immune responses, suggesting that immune processes may be activated in the LGSOC tumor microenvironment. Given the importance of MAPK pathway alterations in LGSOC [19, 31], we focused on MAPK‐related DEGs and identified *C1orf106* as a candidate target for further investigation.

*C1orf106*, also known as innate immune activator (INAVA), has been implicated in various diseases related to tumor growth and progression. However, its role in LGSOC has not been previously reported. In this study, we confirmed that *C1orf106* is highly expressed in LGSOC tissues, suggesting that it may contribute to disease development. Moreover, we observed that high *C1orf106* expression was significantly associated with the absence of residual disease after surgery and with lower BMI (< 25 kg/m^2^).

Mechanistically, *C1orf106* is known to function as an epithelial cell junction protein that interacts with cytohesins through its DUF3338 domain [39]. Downregulation of *C1orf106* has been shown to impair cell–cell junctions. We therefore speculate that reduced *C1orf106* expression in LGSOC may increase the likelihood of residual disease due to weakened cell–cell adhesion, facilitating tumor spread. However, the relationship between *C1orf106* expression and BMI remains unclear, as no prior studies have addressed this association. Additional functional and clinical investigations are warranted to further clarify these findings.

Finally, we examined clinicopathological factors associated with prognosis in LGSOC. Previous studies have shown that prognosis is primarily determined by FIGO stage, residual macroscopic disease, and CA‐125 levels [20, 23]. In our cohort, poor prognostic factors for PFS included large ascitic fluid volume (> 100 mL), positive ascitic cytology, advanced FIGO stage (III–IV), residual macroscopic disease, and low *C1orf106* protein expression. However, multivariate analysis identified advanced FIGO stage (III–IV) as the only independent prognostic factor for PFS. Similarly, advanced FIGO stage (III–IV), large ascitic fluid volume (> 100 mL), positive cytology, and low *C1orf106* expression were poor prognostic indicators for OS, but only advanced FIGO stage (III–IV) remained independently significant. These findings reaffirm that FIGO stage is the most reliable prognostic determinant in LGSOC.

Although *C1orf106* expression was not an independent prognostic factor, univariate analysis and Kaplan–Meier survival curves indicated that higher expression was associated with longer PFS and OS. Thus, *C1orf106* may serve as a supplementary marker for prognosis prediction in LGSOC, although validation in larger patient cohorts is warranted.

The role of *C1orf106* in cancer biology remains poorly understood. Prior studies have reported associations between *C1orf106* expression and tumor aggressiveness or poor prognosis in lung adenocarcinoma [16] and papillary thyroid carcinoma [17]. In contrast, our findings suggest that high *C1orf106* expression is linked to better prognosis in LGSOC. This discrepancy may reflect differences in tumor molecular characteristics and therapeutic strategies across cancer types.

Interestingly, *C1orf106* is not only involved in MAPK signaling but also associated with the activation of ER signaling [40]. While aberrant ER pathway activation typically promotes cancer progression, in LGSOC it has been linked to improved survival in certain patient subsets [40]. Given that LGSOC frequently expresses ER and PR, endocrine therapies are routinely administered after debulking surgery and chemotherapy [41], which may contribute to the favorable prognostic association of *C1orf106* in this context. By contrast, endocrine therapies are not standard in many other cancers, potentially explaining divergent prognostic implications.

Another explanation may lie in the context‐dependent role of *C1orf106* as a regulator of signal transduction. The tumor microenvironment likely shapes its biological effects differently across diseases. For example, *C1orf106* has been implicated in MAPK and NF‐κB pathway activation in inflammatory bowel disease, supporting intestinal immune homeostasis [42], and in dermatomyositis, where it is associated with CD4+ T cell infiltration [15]. Consistent with our enrichment analyses, which identified multiple immune‐related pathways in LGSOC, we speculate that high *C1orf106* expression may promote immune cell infiltration into tumors, contributing to improved prognosis. However, the underlying mechanisms require further study.

In summary, multiple genes and pathways contribute to LGSOC development. Through bioinformatics and experimental validation, we identified *C1orf106* as a novel biomarker highly expressed in LGSOC and demonstrated its potential value in prognosis evaluation. Our findings suggest that *C1orf106* represents a promising candidate for further preclinical studies as both a prognostic marker and a potential therapeutic target.

## Author Contributions

**Feifei Song:** conceptualization, investigation, writing – original draft, methodology, validation, visualization, writing – review and editing, software, formal analysis, project administration, data curation. **Xiaojing Chen:** methodology, validation. **Tao Zhang:** methodology, validation. **Xuedong Tang:** methodology, funding acquisition. **Xiaodong Cheng:** conceptualization, funding acquisition, supervision, resources.

## Disclosure

The authors have nothing to report.

## Ethics Statement

Ethical approval for this study was granted by the Ethics Committee of the Women's Hospital, School of Medicine, Zhejiang University (Approval No. IRB‐20220029‐R).

## Consent

Informed consent was received from all participants.

## Conflicts of Interest

The authors declare no conflicts of interest.

## Data Availability

The datasets generated and/or analyzed during the current study are not publicly available due to ethical and legal restrictions. This retrospective study used clinical data from patients treated at Women's Hospital, School of Medicine, Zhejiang University. Sharing these data outside the institution is not permitted by the ethics committee in order to protect patient confidentiality, and informed consent for public data sharing was not obtained. Researchers who meet the criteria for access to confidential information can request the data from the corresponding author with prior approval from the institutional ethics committee.
